# Surgical clinical trials for HPV-positive oropharyngeal carcinoma

**DOI:** 10.3389/fonc.2022.992348

**Published:** 2022-11-09

**Authors:** Chen Lin, Daniel D. Sharbel, Michael C. Topf

**Affiliations:** ^1^ Department of Otolaryngology – Head and Neck Surgery, Vanderbilt University Medical Center, Nashville, TN, United States; ^2^ Department of Otolaryngology – Head and Neck Surgery, University of Illinois at Chicago, Chicago, IL, United States

**Keywords:** HPV oropharyngeal squamous cell carcinoma (OPSCC), clinical trials, ECOG 3311, ORATOR, PATHOS, EORTC 1420, MC1273/1675, AVOID

## Abstract

The treatment of HPV-positive oropharyngeal squamous cell carcinoma (OPSCC) continues to evolve as multiple ongoing and recently completed clinical trials investigate the role of surgery, radiation therapy, chemotherapy, and immunotherapy. Current trials are investigating transoral robotic surgery (TORS) in treatment de-escalation protocols in an effort to optimize quality of life, while maintaining historical survival rates. The advantage of TORS is its minimally invasive approach to primary resection of the tumor as well as valuable pathologic staging. The ORATOR trial reported poorer quality of life in patients treated with TORS compared to primary radiotherapy though this was not a clinically meaningful difference. The recently published ECOG 3311 trial showed that surgery can be used to safely de-escalate the adjuvant radiation dose to 50 Gy in intermediate-risk patients. In this review, we summarize and discuss the past and current clinical trials involving surgery in the treatment of HPV-positive OPSCC.

## Introduction

Human papilloma virus-associated oropharyngeal squamous cell carcinoma (HPV+ OPSCC) was recognized as a distinct clinical entity over twenty years ago (1). Early molecular studies elucidated the role of high-risk strains of HPV, namely HPV-16, in oncogenesis within Waldeyer’s ring (1, 2). In a landmark 2010 study by Ang et al., HPV status was found to be a strong independent predictor of survival in oropharyngeal squamous cell carcinoma (OPSCC) (3).

Concurrently during this time period, seminal feasibility studies examining the use of the daVinci surgical robot (Intuitive Surgical Inc., Sunnyvale, CA) for transoral surgery of the oropharynx were published by Hockstein, O’Malley, and Weinstein (4–6). O’Malley and Weinstein coined the term transoral robotic surgery, or TORS, in 2006. These initial papers demonstrating feasibility were later supported by studies suggesting oncologic soundness (7–9), as well as excellent functional outcomes including decreased need for feeding tubes and high quality of life (10, 11). This led to the United States Food and Drug Administration (FDA) approval of the da Vinci robot for transoral otolaryngology procedures in 2009.

After the initial identification of HPV+ OPSCC as a distinct disease process, epidemiologic studies have recognized its exponentially increasing incidence in the United States despite declining smoking rates (12–15). In one early study, it was found that HPV+ OPSCC demonstrated a 225% population level increase from 1988-2004 with a concomitant 50% decrease in HPV-negative OPSCC (14). HPV+ OPSCC patients are typically younger and healthier with improved survival compared to patients with HPV-negative, smoking-related head and neck squamous cell carcinoma (14, 16). As this population lives longer, a greater number of patients are developing late-stage toxicities including severe dysphagia after chemoradiation therapy (17, 18).

Given the improved prognosis of HPV+ OPSCC patients, there has been recent emphasis on treatment deintensification to reduce toxicity and improve quality of life among survivors. TORS, with its minimally invasive approach, plays an important role in treatment deintensification for low-risk HPV+ OPSCC patients. Until recently, there has been a paucity of high quality prospective and randomized controlled trials (RCTs) evaluating the role of TORS for the treatment of HPV+ OPSCC (19). This review provides an accessible overview of completed and ongoing RCTs involving TORS for the treatment of HPV+ OPSCC (**Table 1**).

**Table 1:** Surgical clinical trials for HPV-mediated oropharyngeal carcinoma.

	Trial	Phase	Primary location	Status	Eligible patients (AJCC 7th)	Intervention	Primary end point	Results	Other notable findings
Surgical vs nonsurgical trials	ORATOR	II	Canada	Completed	T1-2, N0-2	TORS vs primary RT (70 Gy)	MDADI	80.1 (TORS) vs 86.9 (RT) (p = 0.04)	–
	EORTC 1420	III	Europe	Active	T1-2, N0-1**	TORS/TLM vs primary RT (66-70 Gy)	MDADI	Not available	–
De-escalation trials	E3311	II	United States	Completed	T1-2, N0-2b	TORS/TLM + adjuvant RT (50 Gy vs 60 Gy) for intermediate-risk	2-year PFS	94.9% (50 Gy) vs 96.0% (60 Gy) (p = 0.90)	14% (50 Gy) vs 24% (60 Gy) (p < 0.0001) for >= Grade 3 events
	SIRS	II	Mount Sinai	Completed	T1-2, N0-2b	TORS + adjuvant RT (50 Gy) for intermediate-risk group and TORS + adjuvant RT (56 Gy) + cisplatin for high-risk	PFS, LRF	PFS: 91.3% (low-risk), 86.7% (intermediate-risk), 93.3% (high-risk) LRF: 5 failures	7.4% occult nodal disease in contralateral N0 neck
	ADEPT	III	Washington Univ.	Terminated	ENE with negative margins	Omission of cisplatin for ENE	DFS and LRC	Not available	–
	PATHOS	II/III	United Kingdom	Active	T1-3, N0-2b	TORS/TLM + adjuvant RT (50 Gy vs 60 Gy) for intermediate-risk group and omission of cisplatin for ENE in high-risk group	OS and MDADI	Not available	–
	MC1273	II, single arm	Mayo Clinic	Completed	T1-4a, N0-3	TORS + adjuvant RT (30-36 Gy) + docetaxel	2-year LRC	96.2%	–
	MC1675	III	Mayo Clinic	Completed (not yet published)	T1-3, N0-3	TORS + adjuvant RT (30-36 Gy) + docetaxel vs TORS + adjuvant RT (60 Gy) +/- cisplatin	Adverse events	1.6% (DART) vs 7.1% (SOC) for >= Grade 3 events	2-year PFS in +ENE groups was 78.9% (DART) vs 96.2% (SOC)
	AVOID	II, single arm	Univ. of Penn.	Completed	pT1-2, N1-3	TORS + adjuvant neck RT (60-66 Gy) (primary site spared)	2-year LC	98.30%	Primary site still received mean dose of 37 Gy RT (part of neck treatment field)
	ORATOR2*	III	Canada	Terminated	T1-2, N0-2 (8th edition)	TORS/TLM + adjuvant RT (50 Gy) vs primary RT (60 Gy)	OS	Not available	2 treatment deaths out of 27 surgical patients (hemorrhage and cervical spine osteomyelitis)

*Combination of surgical vs nonsurgical trial with de-escalation protocols.

**T1-2, N0-1 supraglottis and T1-2, N0 hypopharynx also included in trial.

## Clinical trials comparing surgery and primary radiotherapy

### ORATOR

ORATOR (NCT01590355) was the first RCT to compare surgical versus nonsurgical standard-of-care treatment for HPV+ OPSCC (20). The surgical group underwent TORS with neck dissection and adjuvant therapy based on pathology, and the nonsurgical group was treated with primary radiotherapy (RT) with or without chemotherapy. The phase II trial was primarily conducted at Canadian academic institutions with one Australian site from 2012 to 2017. The primary end point was quality of life (QOL) at 1-year based on the MD Anderson Dysphagia Inventory (MDADI) (21).

A total of 68 patients with T1-2, N0-2 (maximum nodal size ≤4 cm) based on AJCC 7^th^ edition staging were enrolled. Those randomized to the radiotherapy group received 70 Gy. Those randomized to the surgical group underwent resection with planned 1 cm margins of the primary tumor. Adjuvant RT at 60-64 Gy was added for pathologic findings: pT3-4, <2 mm margins, any nodal disease, and lymphovascular invasion (LVI). Chemotherapy was added for positive margins and extranodal extension (ENE). Patients with a smoking history were included, but the study did not stratify smokers based on pack years or smoking status.

Contrary to prior retrospective studies (10, 11, 22), the investigators of ORATOR found that primary radiotherapy resulted in higher QOL at 1-year post-treatment (86.9 primary RT vs. 80.1 surgery) (p = 0.04), although this was not deemed clinically significant based on the pre-determined clinically meaningful difference (11, 23–25). Long term QOL at 2 and 3-years showed even smaller differences between the nonsurgical and surgical arms (26). *Post-hoc* analysis of the surgical group revealed similar scores between surgery alone (82.8), surgery + RT (78.5), and surgery + CRT (80.4) (p = 0.76) (20).

The investigators of the study have been widely applauded for their efforts to bring forth the first RCT to compare TORS versus primary RT for the treatment of HPV+ OPSCC. However, the methods and results of the trial have been controversial. One limitation was the modest number of patients. Among 34 surgical patients, only 10 were treated with surgery alone, while 16 received dual modality therapy and 8 received triple modality therapy. Comparing the 1-year MDADI scores of ORATOR and ECOG 3311 (E3311), the surgery + RT scores were very similar between both studies (79.1 at 50 Gy and 78.8 at 60 Gy for E3311 vs 78.5 for ORATOR), but the surgery alone score was over 10 points higher in E3311 (94.7 vs 82.8 for ORATOR) (26, 27). Although a negative margin in this study was >2 mm, surgeons were required to resect the primary tumor with planned 1 cm margins, which can be difficult to achieve in the oropharynx with potentially greater surgical risks and functional implications. While the definition of a negative margin within the oropharynx remains debated, several studies have advocated that a margin as small as 1 mm can achieve high local control (28, 29). Regarding adjuvant treatment, some patients may have been unnecessarily treated based on the study’s pathologic criteria to add RT including for any nodal disease. Another controversial point was the recommendation for tracheostomy. There was an early death due to oropharyngeal bleeding. As a result, the study was paused and authors strongly recommended tracheostomy after the study resumed. Ultimately, 18 of 34 patients had a tracheostomy (26). Tracheostomy is known to significantly decrease patient QOL and is not typically part of the TORS treatment paradigm in the US or other current trials (30). However, the investigators did not find significant differences in QOL between patients treated with or without tracheostomy (26).

### EORTC 1420

The European Organization for Research and Treatment of Cancer (EORTC) 1420 trial (*NCT02984410*), also known as the “Best of” study, is a RCT comparing the “Best of” RT and “Best of” surgery in patients with early-stage oropharyngeal, supraglottic, and hypopharyngeal squamous cell carcinoma (31). This phase III study comparing standard-of-care treatments is currently ongoing at multiple centers across Europe with a total planned enrollment of 170 patients. Accrual began in 2017 and is expected to complete by 2028. Similar to ORATOR, the primary end point is the 1-year MDADI score.

Patients with T1-2, N0-N1 oropharyngeal and supraglottic carcinoma, as well as T1, N0 hypopharyngeal carcinoma based on AJCC 7^th^ edition staging are included. Surgical arm patients can be treated with transoral laser microsurgery (TLM), TORS, or conventional transoral surgery plus neck dissection. Primary RT patients are treated with 66-70 Gy while primary surgery patients are treated with adjuvant RT for close margins <3 mm.

The authors acknowledge the commonalities of this study compared to ORATOR, but point out several key differences. EORTC 1420 includes anatomic subsites outside of the oropharynx, multiple surgical approaches (TORS, TLM, conventional transoral surgery), and a rigorous quality assurance program (32). The study also excludes N2 disease and recommendation for tracheostomy. Of note, the study requires surgeons to return to the operating room for re-resection in the case of positive or close margins. The study protocol does not specify the dose of adjuvant RT in the surgical group and does not specify whether other common pathologic factors (LVI, PNI, number/size of nodes) necessitate adjuvant RT.

## Clinical trials involving surgery for de-escalation of adjuvant treatment

### ECOG 3311 (E3311)

The Eastern Cooperative Oncology Group (ECOG) 3311 (*NCT01898494*) study was a RCT for the de-escalation of adjuvant treatment after transoral surgery (TOS) and neck dissection for HPV+ OPSCC (27). TOS consisted of TORS and TLM. The primary end point was 2-year progression free survival (PFS), and secondary end points included QOL assessed with the MDADI.

This was a phase II, multicenter trial conducted in the United States between 2013 and 2017. A total of 495 patients with T1-2, N0-2b (based on AJCC 7^th^ edition staging) underwent TOS (N = 443 for TORS and N = 41 for TLM) performed by 68 surgeons (33). Based on pathologic findings, patients were stratified into low, intermediate, and high-risk categories that determined their adjuvant treatment. Low-risk patients (T1-2 with negative margins (>3 mm), N0-1 without ENE)) did not receive further treatment (arm A, n = 38). Intermediate-risk patients (T1-2 with close margins (<3 mm) or minimal ENE (≤1 mm) or N2a (1 lymph node > 3 cm) or N2b (2 - 4 nodes ≤6 cm) or with PNI or LVI were randomized to 50 Gy (arm B, n = 100) or 60 Gy (arm C, n = 108). High-risk patients (positive margins or extensive ENE (>1 mm) or >4 nodes) received 66 Gy with cisplatin (arm D, n = 113). The 2-year PFS was >90% across all arms (96.9% for arm A, 94.9% for arm B, 96.0% for arm C, 90.7% for arm D). The 3-year PFS also remained >90% in all arms. There was no difference in 3-year PFS when stratified by smoking history (<10 pack years vs >10 pack years) in arms B, C, and D.

The significance of this trial is in the findings for the intermediate-risk group. The patients treated with dose-reduction adjuvant RT to 50 Gy demonstrated high 2-year PFS similar to standard-of-care adjuvant RT to 60 Gy. The intermediate-risk group included those with ≤1 mm ENE and demonstrated high PFS without chemotherapy. Grade 3-5 toxicities were significantly lower in arm B (50 Gy) at 14% compared to 24% in arm C (60 Gy) (p = 0.03) and 60% in arm D (p < 0.0001). Because of the promising findings of this phase II study, ECOG is planning a phase III trial comparing arm B (TOS + 50 Gy) and standard-of-care primary CRT at 70 Gy.

### SIRS

The Sinai Robotic Surgery trial was a non-randomized study that evaluated the role of de-escalated adjuvant chemoradiotherapy following TORS and neck dissection for treatment of HPV+ OPSCC (*NCT02072148*) (34). Primary endpoints were disease free survival (DFS) and locoregional control (LRC).

This was a phase II, single institution study that enrolled patients from 2014 to 2022. A total of 54 patients underwent TORS and neck dissection for T1-2, N0-2b (based on AJCC 7th edition staging). Of note, all patients underwent bilateral selective neck dissections and ipsilateral lingual artery ligation if tongue was resected. Patients with >20 pack year smoking histories were excluded. Negative margins were defined as >1mm for tonsil and >3 mm for base of tongue.

Low-risk patients (T1-2 with negative margins, no LVI/PNI/ENE) were stratified to surveillance (group 1, n = 24). Intermediate-risk patients (+LVI/PNI, **≤** 1 mm ENE) underwent adjuvant RT at 50 Gy (group 2, n = 14). High-risk patients (positive margins, **≥**1 mm ENE, >3 positive nodes, contralateral/supraclavicular nodes were treated with 56 Gy + cisplatin (group 3, n = 15). Median follow up was 44 months. Disease free survival for all groups was 98.1%, and there were no significant differences in survival curves between groups (p = 0.81). There were 5 locoregional failures: two in group 1, two in group 2, and one in group 3. PFS for low-risk was 91.3%, intermediate-risk was 86.7%, and high-risk was 93.3%. Since all patients underwent bilateral neck dissection regardless of primary site, the authors were able to report on the rate of occult nodal disease in the contralateral cN0 neck - 4 of 54 patients (7.4%). Of these 4 patients, 2 were tonsil and 2 were base of tongue.

### ADEPT

The Adjuvant De-escalation, Extracapsular Spread, P16+, Transoral (ADEPT) study was a prospective trial investigating the role of chemotherapy in patients with ENE who had been treated surgically for HPV+ OPSCC (*NCT01687413*). The primary end point of the study was DFS and locoregional control. All patients were treated with transoral surgery (TORS or TLM) and neck dissection. Those patients with negative margins at the primary site, but with ENE were included in the study, and treated with adjuvant radiation at 60 Gy with or without cisplatin. Patients were given the option to be either randomized (physician chose the study arm) or nonrandomized (patient chose the study arm). The phase III study was held at Washington University School of Medicine and began recruitment in 2013. Unfortunately, the study was closed due to slow accrual and funding issues. Total enrollment reached 42 patients. Results of the study have not been published to our knowledge.

### PATHOS

The Post-operative Adjuvant Treatment for HPV-positive Tumors (PATHOS) (*NCT02215265*) study is an ongoing European RCT of treatment de-intensification for both radiation and chemotherapy in patients treated surgically for HPV+ OPSCC (35, 36). This trial is essentially a combination of E3311 and ADEPT. The primary end point of the phase II study is MDADI at 1-year, while the primary end points of the phase III study are overall survival and MDADI (35).

The phase II portion of the trial was completed at multiple sites in the United Kingdom from 2015 to 2018 with results yet to be published. The trial transitioned directly to Phase III in 2018 with recruitment of 1,100 patients expected to complete by the end of 2022 followed by a 4-year follow up period until 2026. The phase III trial is still conducted predominantly in the UK with additional centers in Europe, Australia, and the US. Patients with T1-3, N0-2b (AJCC 7^th^ edition) or T1-3, N0-1 (AJCC 8^th^ edition) HPV+ OPSCC who undergo transoral surgery (TORS or TLM) and neck dissection are risk stratified based on pathologic findings. Low-risk patients (no adverse pathologic features) will be observed. Intermediate-risk patients (T1-2 with positive PNI/positive LVI/close margins (1-5 mm) or N2a-b) are randomized to 50 Gy or 60 Gy, similar to E3311. High-risk patients (positive margins (<1 mm) or ENE) are randomized to 60 Gy with or without concurrent cisplatin, similar to ADEPT.

### MC1273

MC1273 (NCT01932697) was a phase II single arm, prospective study evaluating a significant dose-reduction in adjuvant radiation (30-36 Gy) after surgical resection (37, 38). The majority of patients (95%) underwent TOS. All patients, intermediate and high-risk, were also treated with docetaxel. The primary end point of the study was 2-year locoregional control (LRC).

This study was performed at the Mayo Clinic (Rochester, MN and Scottsdale, AZ) from 2013 to 2016. Patients with T1-4a, N0-3 HPV+ OPSCC (AJCC 7^th^ edition stage) were enrolled. All patients (n = 80) received de-escalated adjuvant radiation and chemotherapy after undergoing TORS with negative margins and neck dissection. Low-risk patients and those with >10 pack year smoking history were excluded from the trial. Intermediate-risk (n = 37) included ≥T3, PVI/LVI, ≥2 lymph nodes, or any node >3 cm, and this group received 30 Gy adjuvant RT. High-risk (n = 43) was determined by the presence of ENE, and this group received 36 Gy. Patients were treated with twice-per-day fractionated radiotherapy for 2 weeks. The 2-year LRC, PFS, and OS were 96.2%, 91.1%, and 98.7%. The regimen was well tolerated by patients. QOL and swallowing function were mildly improved at post-RT compared to pre-RT, however, swallowing function was not measured prior to surgery.

An interesting aspect of the study was the *ad hoc* financial analysis performed by the investigators. With the reduction in radiation and chemotherapy, the average cost of treatment dropped from $57,845 for standard-of-care adjuvant CRT to $45,884. This represented a 33% reduction in cost of RT and 21% reduction in overall cost.

To date, no other published study has used such an aggressive course of dose-reduced adjuvant radiation. The rationale for using this dose of radiation came from a study on the treatment of anal cancer by Nigro in 1984 (39). He found that primary chemoradiation using 30 Gy with 5-FU and mitomycin was equally effective, if not more, than radical surgery. In a nonsurgical clinical trial, Memorial Sloan Kettering is currently studying the use of primary CRT at 30 Gy vs. standard-of-care 70 Gy in patients identified with (18) F-Fluoromisonidazole [(18) F-FMISO] PET imaging to evaluate tumor hypoxia in lymph nodes (*NCT00606294*, *NCT03323463*) (40). Additionally, the AVOID trial held at the University of Pennsylvania spared adjuvant radiation to the primary tumor site; however, the primary site ultimately received 34-40 Gy as part of treatment for the neck (41).

Of note, MC1273 was a single arm study without a control arm. The authors later compared the study arm of MC1273 to a retrospective matched cohort of patients who received standard-of-care adjuvant CRT (60 Gy and cisplatin) (38). They reported similar survival and recurrence rates. The most significant risk factors were higher T stage, pN2 disease, and ENE. One concern of the MC1273 study was that intermediate-risk patients who would typically receive dual modality therapy were treated with triple modality therapy. Although all patients received chemotherapy, the investigators chose docetaxel over cisplatin due to favorable results of RTOG 0234 showing improved survival and tolerance of therapy (42).

### MC1675

The MC1675 trial, also known as De-escalated Adjuvant Radiation Therapy for HPV associated Oropharynx Cancer (DART-HPV) was a follow up, RCT (n = 194) in which a control arm (n = 115) using standard-of-care adjuvant RT (60 Gy) was compared to the de-escalated therapy group (n = 79) (43). The phase III study was held at the same two Mayo Clinic locations from 2016 to 2020. All patients with T1-3, N0-3 HPV+ OPSCC underwent TORS and neck dissection. Intermediate-risk patients were treated with adjuvant RT to 60 Gy in the control arm, while the study arm received 30 Gy plus docetaxel based on MC1273. The high-risk ENE control arm received 60 Gy plus cisplatin while the study arm received 36 Gy plus docetaxel. The primary end point of the study was adverse events, and secondary end points were OS, LRC, and PFS.

Based off the findings of MC1273, in which 2 out of 3 local failures were in patients with T4a tumors that required multiple intraoperative excisions to achieve negative margins, the investigators excluded T4 tumors and cases requiring >2 intraoperative attempts at negative margins. Moore et al. reported on greater risk of locoregional recurrence in patients who required two or more attempts at negative margins (44). In comparison, EORTC 1420 requires surgeons to return to the operating room for re-resection in cases of close or negative margins (32).

Although results of the study have not been published, findings were presented at the 2021 American Society for Radiation Oncology (ASTRO) meeting. The DART group had significantly less toxicity (1.6% vs 7.1% for ≥Grade 3 adverse events) including far less patients who required a feeding tube (1.6% vs 27.4%). Swallowing function and QOL were also improved in the DART group. Regarding 2-year OS and LRC, both groups were similar. However, 2-year PFS in the DART group was 86.5% while the standard-of-care group was 95.1%. While PFS for both intermediate-risk groups was similar (97.6% for DART and 93.3% for SOC), there were significant differences in PFS within the high-risk ENE groups (78.9% DART vs 96.2% standard-of-care), particularly in patients with pN2 status (AJCC 8^th^ edition). The combination of ENE and pN2 showed significant differences between treatment groups for PFS (42.9% DART vs 100% standard of care) and LRC (77% vs 100%). These results showed that de-escalation therapy can be effective in intermediate-risk patients without ENE. Based on these results, the Mayo Clinic now uses the DART regimen as its standard-of-care in those patients.

### AVOID

The Alternative Volumes of Oropharyngeal Irradiation for De-intensification (AVOID) trial was a phase II single arm, prospective study investigating the sparing of adjuvant radiation to the primary site after surgical resection (41). Patients received standard-of-care 60-66 Gy adjuvant RT to the neck. The primary end point was 2-year local control.

This study was conducted at the University of Pennsylvania from 2014 to 2018. The study enrolled 60 patients with pT1-2, N1-3 (AJCC 7^th^ edition staging) p16+ OPSCC who had undergone TORS plus neck dissection and required adjuvant therapy based on lymph nodes and ENE. Patients had to have negative margins (≥2 mm) without LVI/PNI. Ultimately, all 60 patients had pN2a-3 disease. Adjuvant RT to the ipsilateral neck was delivered at 60-66 Gy while the contralateral uninvolved neck was treated with 54 Gy in all cases. Of note, conventional intensity modulated radiation therapy (IMRT) and intensity modulated proton therapy (IMPT) were both utilized. Patients with ENE received chemotherapy per the discretion of medical oncologists and typically received cisplatin.

The investigators reported high 2-year local control of 98.3% (1 local recurrence out of 60 patients) and OS of 100%. Regarding toxicity, 3.3% (2 patients) required a feeding tube during treatment, but both patients had them removed by study completion, and 3.3% (2 patients) had soft tissue necrosis within the primary resection bed 3 months after radiation. Although the primary tumor was spared of direct adjuvant therapy, the wound bed still received radiation as part of the neck irradiation field including for retropharyngeal nodes. Therefore, the mean dose of radiation received by the primary site was 37 Gy.

Although the primary tumor site in the Mayo Clinic (MC1273 and MC1675) and AVOID trials ultimately received similar mean doses of radiation, there are distinct differences as pointed out by authors of the AVOID trial (37, 41). In the Mayo Clinical trials, the primary site received a homogeneous dose of radiation while the primary site in the AVOID trial received a heterogenous dose because portions of the primary site were located within the field of neck radiation. The Mayo Clinic trials also used hyperfractionated IMRT while the AVOID trial used conventional fractionated regimens and IMPT in 45% of patients. Lastly, the Mayo Clinic trials treated all patients, including intermediate risk, with the addition of docetaxel. Despite these differences in treatment, 2-year local control rates were similarly high in AVOID (98.3%) and MC1273 (96.3%), in which 2 out of the 3 local recurrences were in pT4a tumors (37, 41). Overall, data from these trials is highly encouraging that the primary tumor site, in carefully selected TORS patients, can potentially be treated with significantly lower doses of radiation compared to current standard-of-care 60-66 Gy.

### ORATOR2

ORATOR2 (NCT03210103) was the follow up, phase III RCT to investigate treatment de-escalation in surgical and nonsurgical approaches for HPV+ OPSCC (45, 46). While both ORATOR trials compared primary surgery (TORS) to primary RT, ORATOR used standard-of-care RT doses while ORATOR2 used de-intensified RT doses. The study was again conducted at mainly Canadian institutions from 2018 to 2020. The primary RT arm was treated with 60 Gy based on the results of NRG HN 002 (47). The surgical arm was treated with adjuvant RT to 50 Gy, similar to E3311 and PATHOS (27, 36). Smoking status was stratified based on less than or greater than 10 pack years. The primary end point was OS and secondary end point was QOL based on the MDADI.

Planned accrual was 140 patients, however the trial was terminated in 2020 after 2 treatment-related deaths in the surgical arm. Only 61 total patients had been enrolled. Of 27 patients who underwent surgery, there were 3 total deaths. Of the 2 treatment-related surgical deaths, one death was secondary to oropharyngeal bleeding on postoperative day 4 in a patient with a tracheostomy, and the other death was related to cervical spine osteomyelitis following adjuvant radiotherapy. The third death was due to a myocardial infarction at 8.5 months in a patient who received adjuvant RT and was deemed not to be treatment-related (48).

## Discussion

Several of the above mentioned surgical clinical trials are providing evidence for the de-escalation of adjuvant radiation in carefully selected HPV-positive patients. For intermediate-risk patients, traditional adjuvant doses of 60 Gy may be able to be reduced to 50 Gy based on the results of SIRS and E3311 (27, 34). The Mayo Clinic (MC1273 and 1675) and the University of Pennsylvania (AVOID) trials have all explored further reductions in adjuvant radiation (37, 41, 43). The Mayo Clinic trials delivered 30-36 Gy to the primary site, while the AVOID trial spared the primary site of direct radiation but still received an average of 37 Gy as part of the neck radiation field.

The clinical significance of ENE in HPV+ OPSCC and role for adjuvant chemotherapy remains to be investigated (49). The AJCC 8^th^ edition staging for HPV+ OPSCC no longer accounts for ENE. However, several National Cancer Database studies (50, 51) have shown that ENE positivity results in worse survival in HPV+ OPSCC, while single and multi-institutional studies have not shown a difference (52–55). Based on the degree of ENE, Lewis et al. only found a survival difference in cases where tumor metastasis had completely replaced nodal tissue (grade 4), however, these cases were strongly associated with T4 tumors (52). After controlling for tumor stage in a multivariate analysis, ENE of any degree did not correlate with poorer survival. Several of the clinical trials discussed in this review are investigating the role of chemotherapy in treating ENE. The E3311 and SIRS trials, which stratified patients with ≤1 mm of ENE to intermediate-risk groups, showed that minimal ENE does not require chemotherapy to achieve high survival (27, 34). Going a step further, the PATHOS trial is investigating whether any degree of ENE requires chemotherapy, with the trial expected to conclude in 2026 (35, 36).

Primary surgery has an effective role in both low-risk and intermediate-risk patients (27). In low-risk patients, it can be used as a single modality therapy with high survival and QOL (27, 34, 37, 41, 43), consistent with retrospective and systematic reviews (11, 23, 24). However, there is skepticism toward the role of primary surgery versus primary radiotherapy, particularly after the ORATOR trials (26, 45). One concern of the ORATOR trials has been surgeon experience with TORS and consistency of surgical quality (48). E3311 set high standards for surgical quality assurance by evaluating both quantity and quality of cases performed by each surgeon as part of the credentialing process (33). A committee consisting of 10 experienced head and neck surgeons credentialed each surgeon in the study. Of the 120 surgeons who applied, 87 were approved and 68 ultimately participated in the study (27). Among requirements for credentialing, surgeons had to be associated with an NCI affiliated institution, performed at least 20 TOS cases with pathologic review, and demonstrated proficiency with neck dissection. After approval, surgeons were monitored for continued surgical quality throughout the trial. EORTC 1420 required 25 transoral cases with at least 80% for the oropharynx, at least 5 cases in the past year to be reviewed by the committee, and ≥18 lymph nodes on neck dissection (32). In comparison, the ORATOR and PATHOS trials required 10 and 5 transoral cases, respectively (36, 56). Although ORATOR2 increased their requirements to 20 cases, there were 2 mortalities related to surgery prior to closure of the trial (45). Across ORATOR and ORATOR2, among 61 patients who received surgery, there were 2 deaths related to hemorrhage (3.1%) and 3 total treatment-related deaths (4.9%) (20, 45). In comparison, there was 1 death (0.2%) secondary to hemorrhage among 410 patients in a multicenter TORS retrospective study by de Almeida et al. (9) Similarly in E3311, there was 1 death (0.2%) secondary to hemorrhage among 495 patients (27).

While several of these studies are multi-institutional, all of the current data for de-escalation of adjuvant therapy after surgery for HPV+ OPSCC are phase II studies, with the exception of MC1675. Many oncologists believe these phase 2 trials have not provided enough evidence to change practice patterns. A phase III surgical de-escalation trial will be difficult to complete due to many factors including costs, eligibility, enrollment to reach sufficient power, safety, and time (57).Furthermore, phase II trials such as ADEPT and ORATOR2 did not complete accrual (45). While randomized trials are the gold standard for evaluating surgical treatment, until recently there has been a lack of trials on HPV+ OPSCC. In a study of oncology trials on Clinicaltrials.gov, Menezes et al. reported that only 10% of all trials involved surgery, and less than 1% were randomized surgical oncology trials (58). Despite these challenges, MC1675 was recently completed, and there are currently two Phase III multi-institutional studies underway in Europe: PATHOS (expected completion 2026) and EORTC 1420 (expected completion 2028. The phase III follow-up study to E3311 is being planned, but results of that study are likely more than a decade away.

Finally, circulating tumor HPV (ctHPV) DNA shows promise in both detection and surveillance of HPV-positive oropharyngeal cancer (59–61). By targeting E6 and/or E7 oncogenes, plasma ctHPV DNA can be measured with PCR or next generation sequencing. In the posttreatment period, detection of ctHPV DNA in plasma precedes clinical evidence of recurrence (59). with two consecutive positive tests strongly predicting disease recurrence (60). Clearance kinetics suggest that in low risk patients, ctHPV DNA rapidly decreases to <1 copy/mL by the first postoperative day (61, 62). Therefore, elevated levels are associated with risk of residual disease and could be used as a biomarker for selecting adjuvant therapy in future surgical clinical trial design.

## Author contributions

All authors (CL, DS, MT) contributed to the background research, writing, and editing of this manuscript. All authors contributed to the article and approved the submitted version.

## Funding

Vanderbilt Clinical Oncology Research Career Development Program – K12 NCI 2K12CA090625-22A1.

## Conflict of interest

The authors declare that the research was conducted in the absence of any commercial or financial relationships that could be construed as a potential conflict of interest.

## Publisher’s note

All claims expressed in this article are solely those of the authors and do not necessarily represent those of their affiliated organizations, or those of the publisher, the editors and the reviewers. Any product that may be evaluated in this article, or claim that may be made by its manufacturer, is not guaranteed or endorsed by the publisher.
